# Genistein treatment duration effects biomarkers of cell motility in human prostate

**DOI:** 10.1371/journal.pone.0214078

**Published:** 2019-03-27

**Authors:** Hu Zhang, Ryan Gordon, Wenqi Li, Ximing Yang, Abhinandan Pattanayak, Graham Fowler, Limin Zhang, William J. Catalona, Yongzeng Ding, Li Xu, Xiaoke Huang, Borko Jovanovic, David L. Kelly, Haowen Jiang, Raymond Bergan

**Affiliations:** 1 Division of Hematology/Oncology, Knight Cancer Institute, Oregon Health & Science University, Portland, Oregon, United States of America; 2 Department of Urology, Huashan Hospital, Fudan University, Shanghai, China; 3 Department of Pathology, Northwestern University, Chicago, Illinois, United States of America; 4 Department of Urology, Northwestern University, Chicago, Illinois, United States of America; 5 Department of Medicine, Northwestern University, Chicago, Illinois, United States of America; 6 Department of Gastroenterology, Xiang’an Hospital of Xiamen University, FujianXiamen, China; 7 Department of Preventive Medicine, Northwestern University, Chicago, Illinois, United States of America; 8 Fred & Pamela Buffet Cancer Center, University Nebraska Medical Center, Omaha, Nebraska, United States of America; University of South Alabama Mitchell Cancer Institute, UNITED STATES

## Abstract

**Background:**

Long term dietary consumption of genistein by Chinese men is associated with decreased PCa metastasis and mortality. Short term treatment of US men with prostate cancer (PCa) with genistein decreases MMP-2 in prostate tissue. MEK4 regulates MMP-2 expression, drives PCa metastasis, and genistein inhibits MEK4, decreases MMP-2 expression and dietary dosing inhibits human PCa metastasis in mice. This study examines short- versus long-term treatment effects of genistein in humans and *in vitro*.

**Methods and findings:**

US men with localized PCa were treated on a phase II trial with genistein (N = 14) versus not (N = 14) for one month prior to radical prostatectomy. Prostate epithelial cells were removed from fresh frozen tissue by laser capture microdissection, and the expression of 12,000 genes profiled. Genistein significantly altered the expression of four genes, three had established links to cancer cell motility and metastasis. Of these three, one was a non-coding transcript, and the other two were BASP1 and HCF2. Genistein increased BASP1 expression in humans, and its engineered over expression and knockdown demonstrated that it suppressed cell invasion in all six human prostate cell lines examined. Genistein decreased HCF2 expression in humans, and it was shown to increase cell invasion in all cell lines examined. The expression of MMP-2, MEK4 and BASP1 was then measured in formalin fixed prostate tissue from N = 38 Chinese men living in China and N = 41 US men living in the US, both cohorts with localized PCa. MMP-2 was 52% higher in Chinese compared to US tissue (P < 0.0001), MEK4 was 48% lower (P < 0.0001), and BASP1 was unaltered. Treatment of PC3 human PCa cells *in vitro* for up to 8 weeks demonstrated that short term genistein treatment decreased MMP-2, while long term treatment increased it, both changes being significant (P<0.05) compared to untreated control cells. Long term genistein-treated cells retained their responsiveness to genistein’s anti-motility effect.

**Conclusions:**

Genistein inhibits pathways in human prostate that drive transformation to a lethal high motility phenotype. Long term treatment induces compensatory changes in biomarkers of efficacy. The current strategy of using such biomarkers after short term intervention as go/no-go determinants in early phase chemoprevention trials should be carefully examined.

## Introduction

Prior studies support the notion that the experimental chemopreventive agent, genistein, inhibits prostate cancer (PCa) cell movement in humans and that this in turn inhibits metastatic spread, thereby preventing PCa-specific death [1–3]. As many effects have been ascribed to genistein, it has been considered a non-specific agent. However, its effects are concentration-dependent, and the vast majority of studies use concentrations greater than 3 logs above those associated with dietary consumption. Genistein is found in soy, and individuals consuming soy-based diets have blood concentrations of free genistein in the low nanomalar range [4]. These low nanomolar concentrations of genistein have been shown to bind to and inhibit MEK4 (MKK4/MAP2K4) kinase, thereby inhibiting the following pro-metastatic pathway in human PCa cells: MEK4 → p38 MAPK → MAP2K4 → HSP27 → MMP-2 → cell invasion → metastasis [2, 3, 5–9]. MEK4 has been shown to drive human PCa metastasis in a human PCa orthotopic implantation murine xenograft model [9], and dietary doses of genistein have been shown to inhibit distant metastasis formation in that same model [3]. Using dosing guided by phase I pharmacokinetic studies in US men [10], prospective treatment of men on a phase II trial with genistein for one month prior to radical prostatectomy for localized PCa demonstrated that genistein decreases MMP-2 expression in human prostate epithelial cells [2].

The above studies were undertaken based upon findings in epidemiological studies. Epidemiological studies associate consumption of genistein in the diet with a ~10-fold lower incidence of metastatic PCa in Southeast Asians, only a ~2-fold lower incidence of primary PCa and indicate that immigrants’ risk of PCa death approaches that in the US by one generation [11–16]. These findings are consistent with differences not being entirely genetic and suggest that external factors may affect the biology of metastasis.

While epidemiological studies are able to identify associations, such as between dietary genistein and PCa metastatis, they are limited due to the presence of multiple confounding factors, many of which cannot be identified. The above series of prospective investigations provided corroboratory findings across model systems, inclusive of purified recombinant protein, cell-based signaling pathway, cell-based functional, human xenograft murine models of metastasis, and prospective human phase I and II clinical trials, and together provide a causal mechanism that could in turn explain epidemiological findings.

Although prospective studies support the hypothesis that genistein prevents transformation of human PCa to a lethal high motility phenotype, the prospective phase II study in humans conducted by us had two important limitations: treatment with genistein was only for one month, and we had limited our examination to pathways identified through *in vitro* based investigations. Treatment time is important because chemopreventive agents are typically given over extended periods, and in the particular case of genistein, epidemiologic studies link lower rates of metastasis to lifetime exposure in the diet. Further, while it was rational to examine specific pathways, this strategy does not allow for examination of mechanism of action in an unbiased fashion.

In the current study, we seek to address both of these limitations, and do so through an integrated approach. In this manner we provide two sets of important findings. First, by conducting an unbiased screening for effects of genistein in prostate we demonstrate that it selectively modulates pathways in humans that regulate cell motility. Second, by comparing molecular profiles in prostate tissue from Chinese men living in China (lifetime genistein exposure) to those of US men living in the US (low genistein exposure), we find evidence in humans that biomarker efficacy profile can change as a function of treatment time. This led us to evaluate the effects of treatment time on prostate cells in culture, confirming time-associated changes in biomarker profile. This finding has broad implications for the field of chemoprevention and its reliance on biomarkers in early phase trials where treatment time is short.

## Methods

### Clinical samples

Prostate tissue samples were obtained from PCa patients seen at Northwestern University in Chicago, Illinois, USA, and from patients seen at Huashan Hospital of Fudan University in Shanghai, China. All samples were collected under IRB-approved protocols. Northwestern University and Huashan Hospital IRB and ethics review committees reviewed and approved this research. PDQ Registration No. NCT00058266, IRB approval #: STU00004877. Consent was not obtained because tissues had already been collected, and all identifiers removed. All samples were reviewed by light microscopy, after H&E staining, by a single pathologist specializing in genitourinary cancer (X.Y.). Samples from patients with PCa came from individuals with clinically localized biopsy-proven PCa who had elected to undergo radical prostatectomy. Samples of normal prostate tissue came from patients undergoing prostatectomy for other purposes, including bladder cancer and benign prostatic hypertrophy, or they came from glands bearing PCa, as denoted. Samples from patients with localized PCa who were treated with genistein prior to radical prostatectomy or served as controls came from subjects entered onto a Phase II clinical trial conducted at Northwestern University. This trial was registered with the National Cancer Institute (PDQ Registration No. NCT00058266), and we have previously reported the design, patient characteristics, source of genistein and results associated with this trial [2]. Samples from the genistein trial were fresh frozen; all other samples were formalin-fixed paraffin-embedded (FFPE).

### Assays and materials

Laser capture microdissection (LCM) and RNA extraction were performed as previously described by us [17]. Briefly, the first section in a series was stained with H&E, and the histology was confirmed by a pathologist (X.Y.) specializing in genitourinary cancer. Frozen sections were processed immediately prior to LCM, normal epithelial or cancer cells were micro-dissected on a PixCell II LCM Workstation (Arcturus), total RNA extracted using a PicoPure RNA Extraction kit (Arcturus), RNA integrity analyzed on an Agilent 2100 Bioanalyzer (Agilent Technologies), and RNA liner amplification performed, all as previously described by us [17].

RNA labeling, microarray hybridization, data acquisition and analysis, were performed as previously described by us [17]. Briefly, human oligonucleotide arrays were custom-printed by a dedicated gene array core facility at the Eppley Institute for Research in Cancer and Allied Diseases, University of Nebraska Medical Center, Omaha, NE, constructed from a set of 12,140 sense probes designed by Compugen Inc. (Rockville, MD), manufactured by Sigma-Genosys, Inc. (The Woodlands. TX), scanned on a ScanArray 4000 confocal laser system (Perkin-Elmer), and the initial fluorescence data was filtered and normalized using the QuantArray software package (Perkin-Elmer). When comparing gene expression between different groups, genes were Z-normalized and rank ordered, and outliers identified and selected for further consideration based upon previously reported criteria: Student’s t-test P-value </ = 0.003 between indicated groups, fold difference >/ = 1.8 fold, and gene expression in the top 3% for at least one of the groups, with expression at least 2 fold above background for the other group [17].

Cell culture, manipulation, analysis and associated reagents. The origin, validation and culture conditions of human PC3 and PC3-M metastatic prostate cancer cell lines, 1532NPTX and 1542NPTX immortalized normal human prostate epithelial cell lines, and 1532CPTX and 1542CPTX human prostate cancer cell lines have been described previously by us [18, 19]. Cells were routinely screened for mycoplasma, were drawn from previously validated frozen stocks, and were replenished at intervals not exceeding two months.

Genistein (4, 5, 7-trihydroxyflavone) (Sigma Chemical Co., St. Louis, MO) and transforming growth factor (TGF)β (R&D Systems, Inc., Minneapolis, MN), were prepared and stored as previously described by us [20]. For cell invasion assays, genistein and TGFβ were used at final concentrations of 50 μM and 2 ng/ml, respectively, for 24 h in serum-free media, and control cells were treated with equal volumes of carrier. For cell migration wound closure assays, genistein was used at a final concentration of 10 μM for 3 days, and control cells were treated with equal volumes of carrier. For long term treatment assays, genistein was used at a final concentration of 1 or 10 μM, as denoted, for up to two months, control cells were treated with equal volumes of carrier, and cells underwent twice weekly changes in media, genistein or carrier, as denoted.

Antibodies used to verify protein expression via Western Blot analysis were as follows: anti-BASP1 (Abcam, Cambridge, MA), anti-HCF2 (Affinity Biologicals, Ancaster, Ontario, Canada), anti-glyceraldehyde- 3-phosphate dehydrogenase (clone, CSA-335E; Stressgen, Victoria, AB, Canada). Vectors included β-galactosidase (pCMV-β-gal; Stratagene, La Jolla, CA), brain abundant, membrane attached signal protein 1 (pCMV6-BAPS1, catalog#: SC116221, Origene, Rockville, MD), serpin peptidase inhibitor, clade D (heparin cofactor), member 1 (SERPIND1) (pCMV6-HCF2, catalog# SC120039 Origene, Rockville, MD), vector control (pCMV6-XL5, Origene, Rockville, MD). Constructs were confirmed by sequencing and transient transfection performed with Mirus LT1 transfection reagent (Mirus, Madison, WI) as per the manufacturer’s instructions. siRNA targeting BASP1 (L-019008-00), HCF2 (L-009540-00), and the Non-Targeting Pool (D-001810-10) were transfected with DharmaFECT reagent 3 (Dharmacon, Lafayette, CO), per manufacturer’s instructions.

Quantitative reverse transcription–polymerase chain reaction (qRT/PCR) and data analysis were performed as described previously described by us [2]. For formalin-fixed paraffin-embedded (FFPE) prostate tissue, used to compare gene expression between Chinese and US men, H&E stained slides of prostate tissue 5 microns in thickness were microscopically reviewed by a genitourinary pathologist (X.Y.). Blocks containing >70% tumor cells on both H&E slides prepared from either side of serial 10 micron cuts were selected to support analysis of gene expression in PCa. Normal samples were similarly confirmed free of tumor. For normal and cancer tissue sections, five sections, each of 10 microns in thickness, were cut from the selected blocks. The RNA was isolated from FFPE tissues using the RNeasy FFPE Kit (Qiagen, Valencia, CA, USA), per manufacturer, quantified and purity evaluated on a NanoDrop 2000 (Thermo Scientific, Pittsburgh, PA, USA). Resultant RNA underwent reverse transcription using a High Capacity cDNA Reverse Transcription Kit followed by gene amplification using a TaqMan Universal PCR Master Mix (Applied BioSystems, Foster City, CA, USA), all per manufacturer, on a QuantStudio 6 Flex Real Time PCR workstation, using validated gene-specific exon spanning primers and probe sets (Applied Biosystems, Foster City, CA, USA). Relative gene expression for FFPE samples was calculated using the comparative CT method [21], where each sample was normalized by calculating an average offset of the three housekeeping genes, GAPDH, ATP5E and PGK1 as compared to the global population averages, and the minimum CT value for the population was set as a common reference for each individual gene of interest. Gene expression in fresh frozen prostate tissue and cell lines was normalized to that of GAPDH.

Boyden chamber cell invasion assays were performed as described by us [22], using a 48-well Boyden chamber (product AP48; Neuro Probe, Gaithersburg, MD), Nuclepore Track-Etch Membrane with 8 μm pores (product NC 983–1643; Whatman, Clifton, NJ) that was coated with denatured collagen (product 214340; Difco-Becton Dickinson, Sparks, MD). Wound gap closure assays Wound gap closure assays were performed as described by us [18] with the following modifications, cells were treated with 10 μM genistein prior to the wounding and images were taken at 0 and 16 hrs post scratch.

### Statistical analysis

Differences between genistein-treated and untreated groups were compared by use of the Student’s t-test. Differences in clinical characteristics in the US and China cohorts were evaluated using either the Student’s t-test (age) or a Chi-squared test (stage and Gleason). Differences in the expression levels between patients from US and China were also evaluated by Student’s t-test. Data were considered statistically significantly different for P values of ≤ .05. All statistical tests were two-sided.

## Results

### Genistein selectively affects genes in the prostate of humans that regulate cancer cell motility and metastasis

The yield of RNA from cells captured by LCM is in the low-nanogram range and is insufficient to directly support probing arrays. We have previously developed a method to linear amplify such low-nanogram amounts of RNA and have comprehensively demonstrated that this amplification process does not alter relative gene expression profiles [17].

To evaluate the performance of LCM coupled to linear amplification and array-based gene profiling across multiple patient samples, we compared normal prostate epithelial cells (N = 14 individuals) to PCa cells (N = 10) in prostate tissue from individuals who had undergone radical prostatectomy for localized PCa. Outlier genes are denoted in S1 Fig. Of 3 outlier genes identified, hepsin and microseminoprotein β have already been shown by others to be differentially expressed in PCa [23, 24]. As an additional measure of internal confirmation for our technological approach, we re-cut frozen tissue samples and performed LCM on 8 paired normal/PCa samples. Importantly, for confirmation studies resultant RNA was not linear amplified, but was used to directly support gene specific qRT/PCR analysis. In order to provide enough RNA to support this, 10–20 individual LCM operations were performed on each tissue. We selected 4 genes for analysis: one with expression that was increased in PCa (*hepsin*), one with expression that was decreased (*MT1L*) and two as controls whose expression was unaltered (*PLSCR2* and *SEC14*). As can be seen in S2 Fig, findings between array and qRT/PCR-based measurements of changes in gene expression were concordant for all 4 genes evaluated.

Next we compared gene expression in genistein treated (N = 14 subjects) versus untreated control subjects (N = 14), using tissue obtained as part of a prospective pre-prostatectomy trial. These studies focused upon normal prostate epithelial cells. This population of cells is present in a cancer containing gland, representing an at risk population and constitutes an important target cell population for pharmacologic targeting with chemoprevention agents [2].

**Fig 1:**
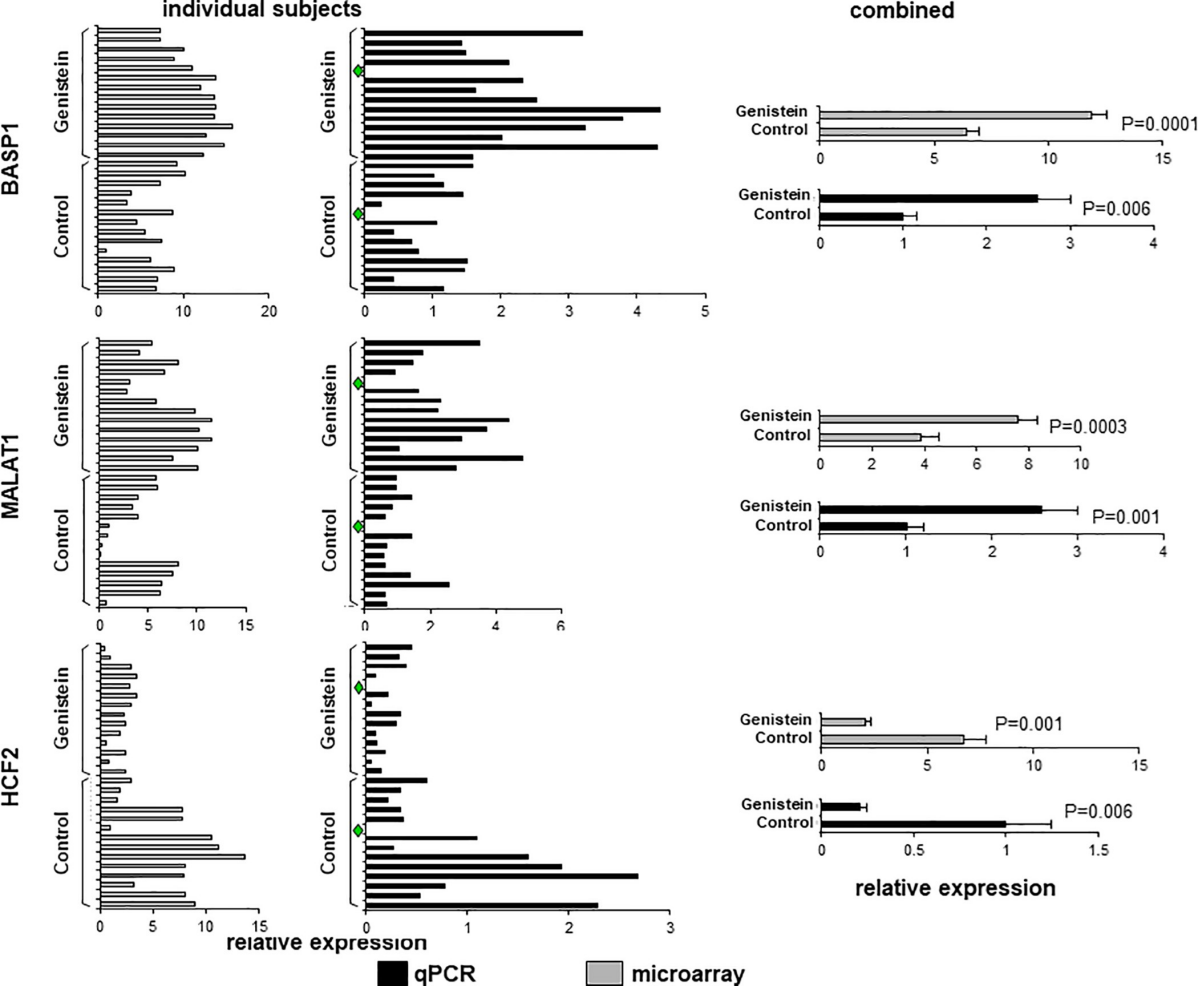
Evaluation of gene expression by qRT/PCR. For each of three genes denoted, the left charts depict mean level of gene expression for each subject by microarray (N = 1) or qRT/PCR (N = 3), as indicated, and as described in S2 Fig. The right charts combine all data for genistein treated or controls. Student’s 2-sided P-values for differences between genistein treatment and controls are depicted. Green diamonds: sample not sufficient to support qRT/PCR analysis.

As can be seen in S3 Fig and depicted in Table 1, four outlier genes, as defined in Methods, were identified (i.e., 0.03% of genes evaluated): metastasis and lung adenocarcinoma transcript 1 (*MALAT1*), heat shock protein 90 (*HSP90*) and brain acid soluble protein 1 (*BASP1*) were increased with genistein, while heparin cofactor II (*HCF2*) was decreased. Three of these genes are closely linked to regulating cell motility and metastasis, and as such were investigated further: HCF2 has been shown to regulate cellular filamentous-actin formation and to promote cell migration [25], BASP1 binds to the actin cytoskeleton and regulates cell motility [26, 27] and MALAT1 is a non-coding transcript associated with metastasis in lung adenocarcinoma [28]. HSP90 is a large chaperone protein, it does affect cell motility and metastasis, but it also affects many cellular functions, and it was therefore not investigated further [29]. A list of all genes where the P value was </ = 0.003 for differences between genistein and control are provided in S4 Fig. Confirmation studies (i.e., re-cutting tissue, LCM and gene-specific qRT/PCR) were performed on *HCF2*, *BASP1* and *MALAT1*, using tissue available from N = 13 genistein treated and N = 13 control subjects. For all three genes evaluated, qRT/PCR assays confirmed array findings, Fig 1. Taken together, these findings demonstrate that genistein selectively affects genes in the human prostate that regulate cell motility and metastasis, and does so in at risk prostate epithelial cells.

**Table 1:** Outlier genes in control and genistein treated subjects.

			mean Z score		
Gene symbol	GenBank accession	P-Value	control	genistein	Ratio (largest/smallest)
BASP1	NM_006317	0.000008	6.40	11.89	1.86
HSP90	D87666	0.001259	4.25	8.02	1.89
MALAT1	AL050210	0.002622	3.88	7.59	1.96
HCF2	NM_000185	0.000754	6.74	2.11	3.19

### Genes altered by genistein in human prostate tissue regulate the motility of human prostate cells in culture

We next sought to assess whether genes altered by genistein in human prostate tissue would affect the motility of human prostate cells. *MALAT1* is a non-coding transcript, and was not investigated further. The functional effects of *BASP1* and *HCF2* were evaluated across a panel of six human prostate cell lines and were done so in the context of engineered changes in gene knockdown as well as overexpression. We have previously extensively characterized this panel of cell lines in a comparative fashion, including their origin, phenotypic and functional characteristics [18, 19]. Together they constitute a representative spectrum of human prostate carcinogenesis, including metastatic PC3 and PC3-M cells, primary localized cancer 1532CPTX and 1542CPTX cells from two different patients, and transformed normal prostate epithelial 1532NPTX and 1542NPTX cells from these same two patients.

Knockdown of BASP1 and HCF2 transcript and protein levels and vector-mediated overexpression of respective transcript and proteins is demonstrated in S5 Fig. In the case of transcripts, siRNA-mediated knockdown decreased expression in control cells as well as in overexpressing cells, did so in all 6 cell lines tested, and did so for both BASP1 and HCF2, S5A and S5B Fig. All changes were statistically significant, except for with PC3 cells, where siRNA-mediated knockdown of HCF in the face of HCF overexpression was only a trend, with P = 0.07. Finally, the siRNA technology deployed used a SMARTpool approach wherein four separate gene specific siRNA species were combined in order to effectively and specifically target a single gene. This strategy decreases off target effects by allowing the concentration of individual siRNA species to be decreased, while allowing for optimized efficacy through combined targeting of different regions of target transcript. It is demonstrated in S6 Fig that individual siRNA species suppress target transcript.

In the case of proteins, siRNA-mediated knockdown decreased expression in control cells as well as in overexpressing cells, did so in all 6 cell lines tested, and did so for both BASP1 and HCF2, with one exception, S5C and S5D Fig. The one exception related to BASP1 in 1532NPTX cells. When BASP1 was overexpressed in 1532NPTX cells, the resultant signal on Western blot was so high that exposure time was limited, precluding detection of protein in control cells, and also precluding documentation of further knockdown by siRNA. It should be noted, however, that siRNA was highly effective in BASP1 overexpressing 1532NPTX cells, S5C Fig, that there was significant suppression of BASP1 transcripts in control 1532NPTX cells by siRNA, S5A Fig, and that BASP1 protein was in fact expressed in native 1532NPTX cells, S5E Fig. Specifically, recognizing that low protein levels are seen in control lanes for all 6 cell lines tested, for the same reason (i.e., limitations in exposure time due to high protein levels in vector-mediated overexpression cells), expression of both BASP1 and HCF2 proteins in native cells was evaluated by Western blot, demonstrating their presence in all 6 cell lines tested, S5E Fig.

**Fig 2:**
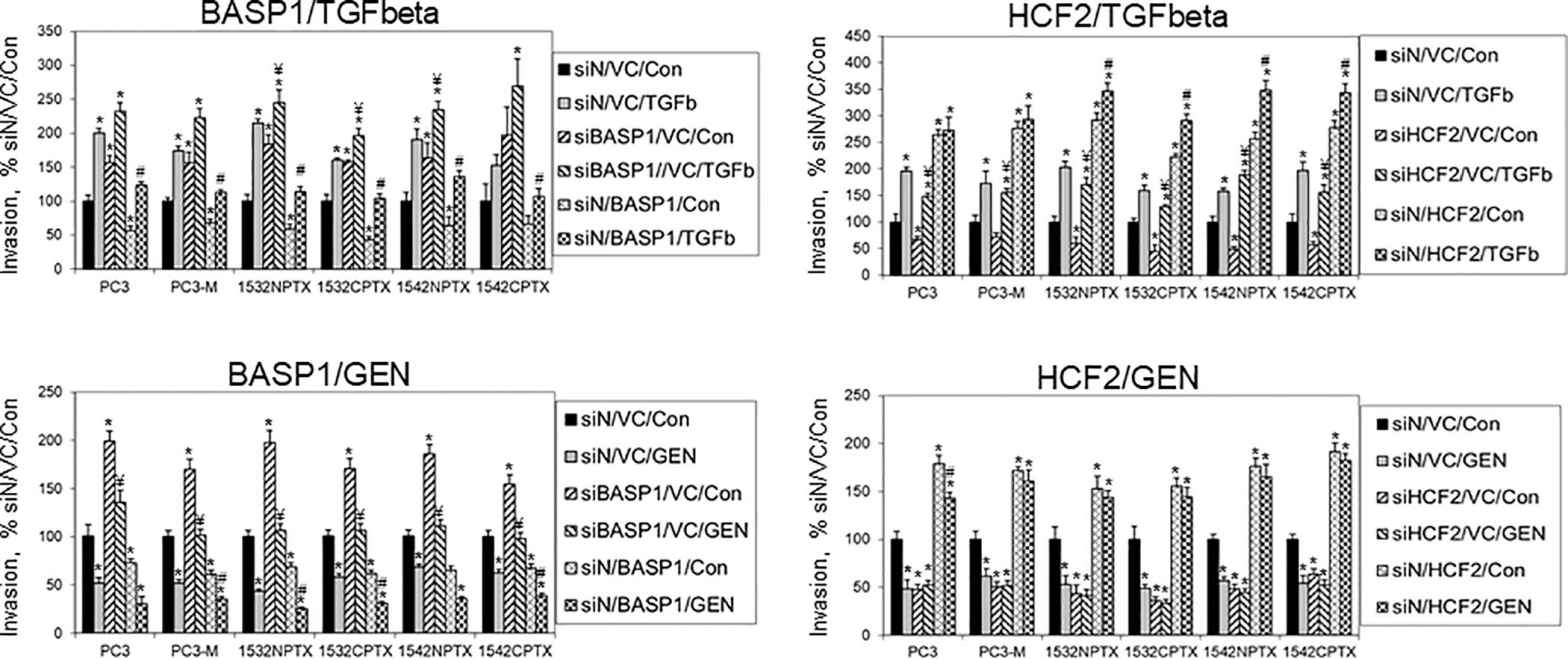
The effect of BASP1 and HCF2 on human prostate cell invasion and genistein efficacy. Each of six different human prostate cell lines, as denoted, were transfected with siRNA to BASP1, HCF2 or non-targeting siRNA (siN), overexpressing vector, vector control (VC), pre-treated with genistein or TGFβ, and cell invasion measured. Data are the mean ± SEM cell invasion (N = 4 replicates), expressed as percent of control cells (siN/VC/Con, not treated [vehicle only]).

Knockdown of BASP1 increased invasion in all cell lines tested, while its overexpression suppressed it, Fig 2. The opposite effect was observed with HCF2: knockdown decreased invasion, while overexpression increased it. We next evaluated whether changes in BASP1 or HCF2 would alter the effect of treatment with genistein or with TGFβ. Genistein is known to inhibit cell invasion, while TGFβ increases it, and this was seen in all cell lines tested in the current study, Fig 2. In the case of BASP1, both genistein and TGFβ retained their efficacy irrespective of changes in BASP1 expression. Specifically, in the case where knockdown of BASP1 increased invasion, genistein significantly decreased invasion in 6 of 6 cell lines. While TGFβ did further increase invasion in all 6 cell lines, this effect was statistically significant in 3; possibly reflecting the already high motility of this engineered phenotype. Where overexpression of BASP1 decreased invasion, TGFβ significantly increased invasion in all 6 cell lines. While genistein did further decrease invasion in all 6 cell lines, this effect was statistically significant in 4; possibly reflecting the already low motility of this engineered phenotype.

In contrast to the situation with BASP1, genistein did not exert any additional effect when HCF2 expression was altered, with the exception of a small but significant decrease in PC3 cells with HCF overexpression, Fig 2. However, the effects of TGFβ remained relatively intact irrespective of HCF2 expression status. Specifically, with HCF2 knockdown, TGFβ significantly increased invasion in 6 of 6 cells tested. With HCF2 overexpression cell invasion increased, and was significantly further increased by TGFβ in 4 of 6 cell lines evaluated. However, in two cell lines TGFβ failed to further increase cell invasion, possibly reflecting the fact that these cells were already at maximum motility under the influence of HCF2 overexpression. Finally, additional control studies were conducted to demonstrate that siRNA-mediated knockdown of BASP1 or of HCF2 were reversed by overexpression with their respective vectors. These studies were conducted in PC3-M cells treated with genistein, TGFβ or vehicle control, and examined effects upon cell invasion, S7 Fig.

These findings demonstrate that genes altered in prostate tissue by genistein regulate the invasion of human prostate cells *in vitro*. Further, they demonstrate that BASP1 suppresses cell invasion, and that HCF2 increases it. Moreover, they indicate that the changes induced by genistein in prostate tissue act to inhibit invasion. Specifically, genistein-induced increases in BASP1 and decreases in HCF2 in prostate tissue are both effects that act to decrease cell invasion. Finally, these studies demonstrate that engineered changes in HCF2 expression negate genistein’s pharmacologic action.

### Comparisons of pathway relevant gene expression in Chinese versus US prostate tissue

With the pre-prostatectomy study described above the average treatment time was four weeks. This is of relatively short duration and it is not atypical for early phase chemoprevention trials, especially window-of-opportunity trials. Epidemiological studies support the notion that lifetime exposure to high dietary genistein by Southeast Asians, including Chinese men, is associated with lower levels of PCa metastasis [11–16]. We therefore hypothesized that if the association between high dietary genistein and low rate of metastasis observed in epidemiologic studies was causal, then changes in pathways linked to transformation to a high motility metastatic phenotype should be evident in prostate tissue of Chinese men as compared to tissue from US men.

To examine this hypothesis we collected prostate tissue from Chinese men living in China and US men living in the US, and measured the expression of a panel of genes selected based on their relevance to genistein’s action. Chinese men living in China consume a diet high in genistein and therefore have been exposed to genistein for a lifetime. Conversely, US men living in the US consume a low-genistein red meat-based diet. Cancer tissue was collected from N = 38 Chinese and N = 41 US men with clinically localized PCa who underwent radical prostatectomy. Their clinical characteristics are shown in Table 2. There were significant differences between the Chinese and US cohorts. Significant differences include Chinese men being 7 years older than US men and US men having a higher stage than Chinese men. Also, there was a statistically non-significant trend of higher Gleason scores in US men. In addition to collecting cancerous tissue, normal prostate tissue was collected. For Chinese men normal tissue was collected from N = 33 individuals, with N = 17 coming from normal tissue present in cancer containing organs and N = 16 coming from organs resected for benign prostatic hypertrophy (BPH) without cancer. For US men normal tissue was collected from N = 21 individuals, all coming from cancer containing organs.

**Table 2:** Characteristics of patients from China and the US.

Characteristic		Chinese		US		p
Patients, No.		38		41		
Mean age, y (range)		69	(56–83)	62	(51–76)	<0.01^§^
Clinical stage, No. (%)						<0.05^¥^
	T1 & T2	28	(74)	19	(46)	
	T3 & T4	10	(26)	22	(54)	
Gleason score, No. (%)						0.06^¥^
	5 & 6	13	(35)	11	(27)	
	7	15	(41)	10	(24)	
	8	6	(16)	7	(17)	
	9 &10	3	(5)	13	(32)	
	unknown	1	(3)	0	(0)	

§ 2-sided student's t test p value

¥ Chi-squared p value

Two sets of genes were evaluated in tissue by gene-specific qRT/PCR. The first set relates to genes that have been implicated in genistein’s regulation of prostate cell motility and metastasis and included *MEK4*, *MMP-2* and *BASP1*. MEK4 was evaluated because it has been shown to drive human PCa metastasis [9], and is the pharmacologic target for genistein-mediated decreases in MMP-2 and inhibition of human PCa cell invasion [2]. There was not enough prostate tissue from the short term treatment trial to support a qRT/PCR-based analysis of *MEK4* gene expression. However, *MEK4* was evaluated on the gene array, all genes evaluated to date by qRT/PCR confirm array findings, and therefore we consider array findings informative. Based on array findings, *MEK4* expression ranked in the top 32% of all genes evaluated (i.e., it was robustly quantified by array data), it was only 11% higher in genistein treated subjects, and this difference was not statistically significant (student’s 2-sided t-test P value = 0.72). Increased MMP-2 protein expression in human PCa has been shown by several groups to increase cell invasion *in vitro* and to portend development of metastasis in humans [30]. Further, it was previously shown by us to be suppressed by genistein in human PCa cells *in vitro* and in prostate tissue after short term genistein treatment, in the same cohort examined above [2]. *BASP1* was evaluated because it was upregulated in prostate tissue after short term genistein treatment and was shown above to suppress human PCa cell invasion.

While *HCF2* was also implicated in studies described above, it was not measured due to technical limitations. Specifically, fresh frozen prostate tissue was used in the phase II pre-prostatectomy study and supported the measurement of *HCF2* gene expression by qRT/PCR. However, FFPE tissue was used when comparing Chinese and US cohorts. We were unable to develop an acceptable qRT/PCR assay for *HCF2* gene expression in FFPE tissue, despite extensive effort. The second set of genes evaluated constitutes a panel of control genes and included *GAPDH*, *ATP5E* and *PGK1*. Control genes were used to normalize the expression of genes in the first set. We recognize that many investigations rely upon a single control gene, prototypically GAPDH, in an effort to preserve limiting amounts of tissue. We elected this more rigorous approach in recognition of the facts that the tissue being compared comes from completely different regions of the world, and our goal of obtaining robust findings.

**Fig 3:**
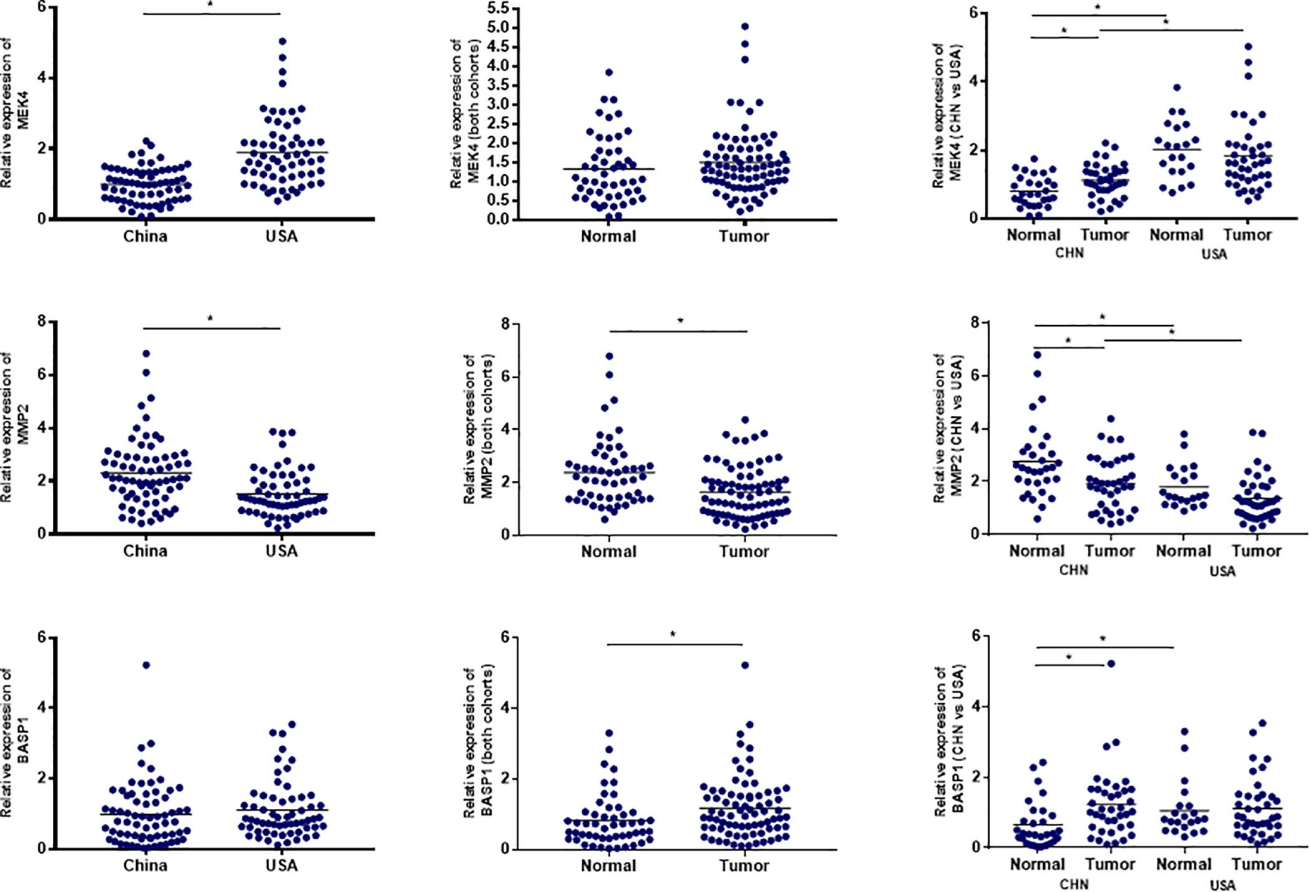
Expression of genes linked to regulation of genistein efficacy in prostate tissue from Chinese and US men. The expression of MEK4, MMP-2 and BASP1 was measured by qRT/PCR in normal and cancer containing prostate tissue from N = 38 Chinese and N = 41 US men. Data are relative expression values, normalized to GAPDH, ATP5E and PGK1, for individual patients, with * denoting Student’s t-test P-values </ = 0.05 between the indicated cohorts.

As depicted in Fig 3, several statistically significant differences between Chinese and US cohorts were identified. When considering these findings, it is very important to consider that tissues were not microdissected, and therefore analysis of any given tissue type, eg., cancer, consists of a heterogenic mix of multiple cell types. We therefore consider the more meaningful comparisons to be between Chinese and US, for which this study was designed. We consider comparisons between normal and cancer tissue to be less informative, but none-the-less, do present them.

Comparing Chinese to US tissue, the most notable difference is in the expression of *MEK4*, where a 1.92 ± 0.12 (mean ± SEM; P < 0.0001) fold decrease in MEK4 expression is observed in Chinese compared to US prostate tissue. MEK4 is a known pharmacologic target for genstein-mediated inhibition of human prostate cell motility [2], and MEK4 is a known driver of human PCa metastasis [9]. Considering only normal or only cancer tissue, *MEK4* expression is 2.50 ± 0.22 (P < 0.007) lower normal tissue and 1.63 ± 0.14 (P < 0.001) lower in cancer tissue, in Chinese compared to US.

With respect to *MMP-2*, changes tended to be opposite of those observed with *MEK4*. Specifically, when all Chinese tissue was compared to all US tissue, *MMP-2* expression was 1.52 ± 0.11 (P < 0.0001) higher in Chinese compared to US. Considering only normal or only cancer tissue, *MMP-2* expression is 1.53 ± 0.13 (P < 0.005) higher normal tissue and 1.41 ± 0.12 (P = 0.05) higher in cancer tissue, in Chinese compared to US.

With *BASP1*, when all Chinese tissue was compared to all US tissue, there was no significant difference. Considering only normal or only cancer tissue, *BASP1* expression is 1.63 ± 0.25 (P < 0.007) lower in normal tissue and not significantly different cancer tissue, in Chinese compared to US.

For non-cancer tissue, from the Chinese cohort N = 17 came from normal tissue present in cancer containing organs and N = 16 came from organs resected for BPH, while from the US cohort all normal tissue was from that present in cancer containing organs. We therefore compared all Chinese to all US tissue, excluding BPH tissue. Exclusion of BPH tissue did not alter overall findings. Specifically, in Chinese tissue MEK4 was lower (P < 0.001), MMP-2 was higher (P < 0.001) and there was no difference in BASP1 expression (P = 0.94), compared to that in US tissue.

Considering comparisons between normal and cancer tissue, several statistically significant (i.e., P </ = 0.05) differences were identified. For MEK4, it is 1.40 ± 0.1 fold higher in cancer compared to normal in Chinese tissue. For MMP-2, it is 1.46 ± 0.77 fold lower in cancer compared to normal for Chinese and US tissues combined, and it is 1.44 ± 0.12 fold lower in cancer compared to normal in Chinese tissue. It should be noted that while prior studies of MMP-2 protein described increases in cancer compared to normal for human prostate, studies which measure transcript levels, as we are currently doing, have shown opposite findings [31]. For BASP1, it is 1.44 ± 0.12 fold higher in cancer compared to normal for Chinese and US tissues combined, and it is 1.85 ± 0.24 fold higher in cancer compared to normal in Chinese tissue.

### Long term genistein treatment induces compensatory changes in biomarker profiles

The finding that MEK4 was decreased in Chinese was not surprising. MEK4 is a pharmacologic target of genistein, Chinese are exposed to high dietary genistein, and a logical cellular response would be to alter signaling pathways in a compensatory fashion. However, the finding that MMP-2 expression was increased in Chinese and varies inversely with that of MEK4 was unexpected. As a protease, MMP-2 has been linked to increased PCa cell motility and metastasis. Further, prior findings demonstrated that MEK4 positively regulated *MMP-2* expression *in vitro*, that inhibition of MEK4 *in vitro* decreased *MMP-2* and that short term treatment of men with PCa with genistein decreased *MMP-2*, while having no effect on *MEK4*. These new findings suggested the notion that prolonged treatment with a chemopreventive agent may induce compensatory changes that alter the biomarker response profile.

**Fig 4:**
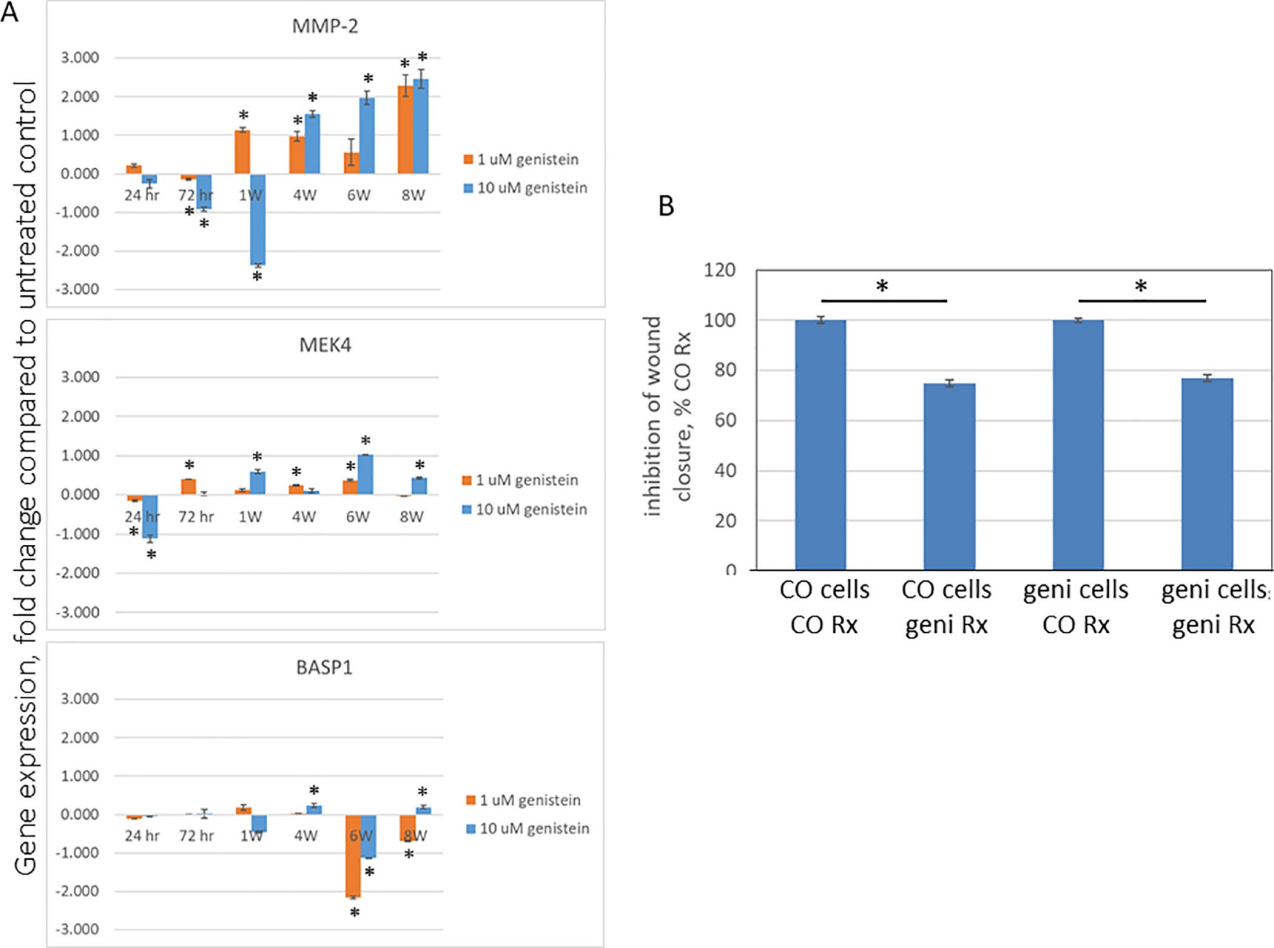
Long term genistein treatment alters biomarker response profile, but not therapeutic responsiveness. PC3 cells were cultured for the indicated times in the presence of either 1 or 10 μM genistein, or equivalent vehicle for controls. A) Effect on gene expression. Cells were harvested at the indicated time points, expression of the indicated genes measured by qRT/PCR, normalized to that of GAPDH, and expressed as the fold difference of genistein treated to respective control cells. Data are the mean ± SEM of N = 3 replicates; with similar results seen in a separate experiment, also N = 3. * denotes Student’s t-test P-value </ = 0.05 between genistein and control cells at a given time point. B) Effect on cell migration. PC3 cells that had been treated with 10 μM genistein for 8 weeks (geni cells), and vehicle control cells (CO cells), were cultured for 3 days in the absence of genistein/vehicle (i.e., a 3 day washout), treated with 10 μM genistein (geni Rx) or vehicle control (CO Rx) for 3 days, and closure of wound gap measured, and expressed as a percentage of CO Rx for each of the cell types. Data are the mean ± SEM of N = 50 replicates; with similar results seen in a separate experiment, also N = 50. * denotes Student’s t-test P-value </ = 0.05 between the indicated conditions.

We investigated this hypothesis by treating PC3 cells with 1 or 10 μM genistein, or with vehicle for controls, for up to 8 weeks, and serially measured the expression of *MEK4*, *MMP-2* and *BASP1* transcript levels by qRT/PCR, Fig 4. As previously reported, genistein significantly decreased MMP-2 expression in a concentration-dependent manner at 72 hrs (i.e., short term exposure). However, between 1–4 weeks this trend reversed, MMP-2 expression increased compared to controls and at 8 weeks *MMP-2* expression remained significantly elevated, was greater than twice that of control cells, and this was seen with both 1 and 10 μM treated groups. In contrast, with either *MEK4* or *BASP1*, the magnitude of the changes were small, dose-dependency was erratic, and no clear trends were apparent. These findings demonstrate that prolonged treatment with genistein alters it’s biomarker response profile, and that of MMP-2 in particular.

We next examined whether cells treated long term with genistein would still be responsive to genistein’s therapeutic anti-motility action. As we had previously demonstrated that genistein’s anti-invasion action was primarily due to inhibition of cell migration [20], we measured effects on cell migration, and did so with a wound healing assay. PC3 cells that were treated for 8 weeks with genistein, or vehicle control, were given a 3 day washout period, then treated with genistein or vehicle, and inhibition of wound gap closure was measured, Fig 4. It can be seen that genistein significantly inhibited wound closure in both long term genistein treated and control cells, with the degree of inhibition being essentially identical. These findings demonstrate that after long term treatment with genistein, cells retain their therapeutic responsiveness, but exhibit a differential biomarker profile. Further, our findings using cells *in vitro* emulate findings in humans in that in both models short term genistein treatment decreases MMP-2, whereas long term exposure increases MMP-2.

## Discussion

We deployed an upfront screening strategy designed to probe for effects induced by an experimental chemopreventive agent on at risk target tissue in humans. The screen used gene expression profiling, the experimental chemopreventive agent was genistein and the at risk tissue was histologically normal prostate epithelial cells residing in a cancer-containing organ. Through this strategy we demonstrated that genistein selectively modulates genes that regulate cell motility in at risk human prostate tissue.

These findings provide additional support for epidemiological studies that associate dietary genistein with decreased PCa metastasis and mortality [11–16]. They also serve to corroborate prior prospective pre-clinical and clinical studies from a functional standpoint in that current and prior studies demonstrate that genistein inhibits prostate cell motility [1–3, 7, 9, 12]. A point of difference is that in the current study, our unbiased analysis of genistein’s effects on human prostate did not identify MMP-2, or other members of the MEK4 pro-motility pathway. A central reason for this relates to the fact that when screening over 12,000 genes, we built in highly rigorous selection criterion [17]. Our purpose was to reduce false positives. This strategy was successful in that the genes we found altered by genistein in human tissue were confirmed to regulate human PCa cell motility *in vitro*. We recognize the inherent drawback of our approach is increased false negatives. This is the case with MMP-2. Our prior *in vitro* findings led us to specifically test MMP-2 in humans, and we did demonstrate in prior reports that it does decrease in prostate tissue after short term treatment of men [2].

Of high importance is the proof-of-principle demonstration that by undertaking an upfront screening strategy in relevant target tissue after prospective treatment of humans with an experimental chemopreventive agent it is possible to rapidly identify a mechanism of action operative in humans relevant to cancer prevention. The potential impact of this is highlighted by considering the fact that for many experimental chemopreventive agents, the literature links each to several different mechanisms of action. As such it is difficult to prioritize the mechanisms that are operative in humans. Multiple factors contribute to this issue, including inherent limitations in preclinical models, use of high concentrations in pre-clinical testing, multiple pharmacologic effects induced by many agents, as well as unknown effects of metabolites.

The current approach offers a relatively efficient pathway for identifying mechanisms operative in human target tissue for experimental chemopreventive agents. As described above we deployed gene arrays, however, this approach could be readily adapted to many other modes of screening. We consider key elements to success to relate to several factors, including choice of target tissue and how that it was harvested. In the current study, we used laser capture microdissection to allow recovery and analysis of a relatively pure population of cells. Further, we focused our analysis on an at risk cell population, in this case normal prostate epithelial cells adjacent to cancer cells. An equally important feature was study design, including choice of agent, dose, randomization, and utilizing a pre-surgical population, thus providing ample high-quality tissue for probing.

A related important concept was that we began with screening approaches in humans, which then informed the design of cell culture-based *in vitro* studies. In this particular case, we demonstrated that BASP1 suppressed and HCF2 increased cell invasion. While additional studies focusing on these protein products need to be conducted at the pre-clinical level in order to gain a deeper understanding of how they may interface with other identified pathways that regulate cell motility, they can be conducted with the confidence of knowing that they can be pharmacologically modulated in humans. Further, by demonstrating that engineered changes in HCF2 expression mitigated additional pharmacologic efficacy by genistein, we thereby identified a high priority pathway regulating genistein action. Finally, it was important that our *in vitro* analysis included transformed normal prostate epithelial cell lines, which represent a model of at-risk pre-cancerous cells, and as such, relevant target cells for chemopreventive agents.

A third important achievement in this study relates to a comparative analysis of tissue from Chinese and US men. The differential demographics of cancers across the globe are frequently cited as important learning opportunities. Unfortunately, those opportunities tend to be limited. While the study of incidence and mortality rates across different regions is of very high value for identifying differences in disease occurrence and outcome, the associated confounding factors tend to preclude more definitive statements. Multiple barriers exist when one seeks to acquire and analyze tissue samples across regions for the purpose of examining specific mechanisms of disease. In carrying out the current study, we demonstrated our ability to overcome these barriers. There are many confounding factors that are in play when one just considers a comparison of tissues harvested from men in China to those harvested from men in the US, and this consideration holds true for the current study. It was therefore of high importance that we then used findings from this comparative tissue analysis to inform the design of prospective studies on cells *in vitro*. The findings from those studies corroborate those from the comparative tissue analysis, and thereby provide data that serves to mitigate the impact of uncontrollable and unknowable confounding variables. These findings notwithstanding, our current findings provide one possible explanation for differences between Chinese and US prostates.

Our comparative analysis of Chinese and US prostate tissue demonstrated decreased levels of *MEK4* expression in Chinese prostate compared to that of the US. The magnitude of this decrease was not small, i.e. ~50%, and was statistically significant. This finding was pathway relevant, as MEK4 has been shown to be a pharmacologic target of genistein, and for genistein-mediated inhibition of motility and metastasis in particular [2, 3]. Further, MEK4 has been shown to be a driver of metastasis for human PCa [9]. This finding in Chinese men supports the notion that long-term exposure to genistein leads to down regulation of its pharmacologic target. The concept that long-term exposure leads to compensatory changes is further supported by a consideration of MMP-2 expression. It was increased in Chinese men, compared to US men. This was surprising in that a relatively comprehensive series of studies conducted by us demonstrated that MEK4 drives increases in MMP-2 and in Chinese men, MEK4 was in fact lower compared to US men. Recognizing that all of our prior studies were conducted after short term genistein exposure, we went on to demonstrate that after long term genistein treatment, MMP-2 in fact increases. Together, these findings demonstrate that with prolonged treatment with genistein, cells undergo compensatory changes in pharmacologically relevant target pathways. This results in an altered biomarker response profile. In this particular instance, we demonstrated that this situation is associated with retention of therapeutic efficacy, at least in the models we examined.

There are inherent challenges in ascertaining the biology operative in humans exposed to an agent at low doses over many years. The investigations conveyed in the current manuscript encompass analysis of effects in humans after short-term and long term exposure, build upon a robust set of pre-clinical and clinical studies that provide concordant findings across model systems that span human recombinant protein in vitro, cell line, animal model and prospective human trials, and they encompass analysis after short-term and long-term treatment of human prostate cancer cells in culture. As such, they serve to provide an integrative framework for understanding how an agent with biologically important properties is acting in humans. By demonstrating differences in effects achieved after short term and long term exposure in humans, and then going on to demonstrate those same changes under rigorous cell culture conditions in the laboratory, the current study serves to provide an integrative framework for understanding previous findings, by us and others. Importantly, it also provides a framework for the design of future studies.

Taken together, our findings also raise a cautionary concern for the design of prospective intervention biomarker-based chemoprevention clinical trials. In the field of chemoprevention, it is current practice to conduct short term intervention trials in which central endpoints are putative biomarkers of therapeutic efficacy. The effect of therapy upon such biomarkers is then used to determine further development in a go/no-go type of fashion. Our current findings demonstrate that different effects upon individual biomarkers are observed after short-term exposure to a chemopreventive agent, as compared to that seen after long-term exposure. This appears to be the case with genistein in the current study. In fact, we had previously reported changes in the expression of pathway-relevant proteins in human PCa murine xenografts after several weeks of treatment with dietary amounts of genistein [3]. At that time, we had raised the prospect of long-term compensatory changes. Findings from the current study further support this notion. They raise the prospect that our findings in the particular case of genistein are in fact more generalizable to other experimental chemopreventive agents. This notion is further supported by the fact that compensatory responses to therapeutics for advanced cancer are an expected reality. Given the emphasis placed upon using short term biomarker response information to guide further development of experimental chemopreventive agents, and the associated commitment of resources, findings from the current study provide evidence that this strategy should be closely examined. It will be important for future studies to examine the mechanisms underlying the changes in biomarker responses induced by genistein treatment.

There are inherent limitations to the current study. There were differences between the clinical characteristics between the Chinese and US cohorts. These were statistically significant for age and stage, and trending for Gleason score. This reflects the facts that there are inherent differences in health care delivery between China and the US. It is possible that these factors contributed to differences in molecular profiles identified by us between cohorts. However, an analysis within each cohort for effects by these clinical features upon gene expression failed to yield effects (not shown), thereby serving to mitigate this concern. Another limitation relates to the biological meaning of downregulated *MEK4* and upregulated MMP-2 in the Chinese cohort. While we attributed this to mean downregulation of a pro-motility pathway, it is also possible that other compensatory changes within the regulatory pathways counteract this, and the sum effect is nil. Our follow-on prospective *in vitro* studies of long term genistein treatment provide evidence that therapeutic efficacy is retained.

In summary, we demonstrated that prospective treatment of US men with genistein selectively targeted genes in at risk prostate tissue that regulate human prostate cell motility. Further, the changes in *BASP1* and *HCF2* expression observed in humans were subsequently shown to regulate cell motility *in vitro*. These findings supported the value of screening in at-risk tissue after prospective intervention with experimental chemopreventive agents in humans. We then went on to examine pathway-relevant effects in Chinese and US cohorts. These studies demonstrated downregulation of the pharmacologic target of genistein in Chinese men, who experience lifetime exposure to dietary genistein. They also indicate that biomarker expression can change as a function of treatment time.

## Supporting information

S1 Fig Differences in gene expression between cancer and normal epithelial cells in human prostate tissue. (PDF)

S2 Fig Evaluation of gene expression by qRT/PCR. (PDF)

S3 Fig Differences in gene expression in normal epithelial cells in human prostate tissue between genistein treated and control subjects. (PDF)

S4 Fig List of all genes with P </ = 0.003 for the difference between genistein and control. (PDF)

S5 Fig Effect of BASP1 and HCF2 knockdown and overexpression on respective transcript and protein levels. (PDF)

S6 Fig Effect of individual siRNA species on target gene transcript levels. (PDF)

S7 Fig The effect of BASP1 and HCF2 on human prostate cell invasion and genistein efficacy. (PDF)
