# Supplementary material for: Genistein treatment duration effects biomarkers of cell motility in human prostate
Source: PLoS One. 2019 Mar 27;14(3):e0214078. doi: 10.1371/journal.pone.0214078 (PMC6436751; doi:10.1371/journal.pone.0214078)
Supplement: S1 Fig — (PDF) [file pone.0214078.s001.pdf]

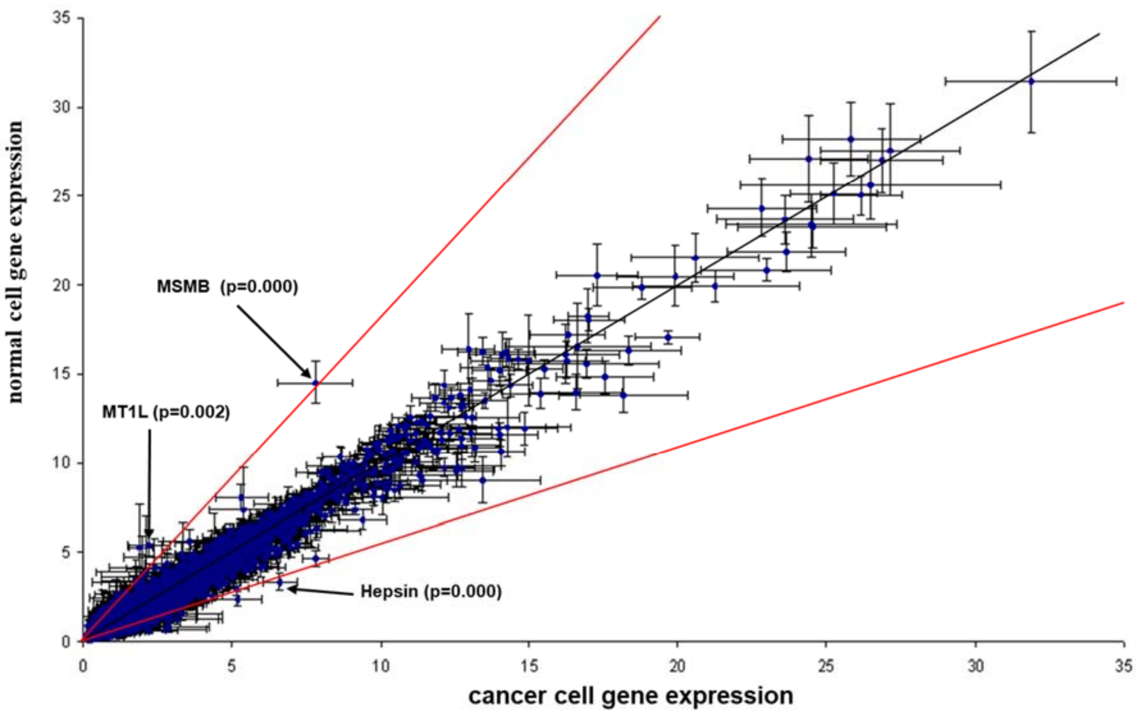

S1 Fig. Differences in gene expression between cancer and normal epithelial cells in human prostate tissue. Normal prostate epithelial cells from N = 14 individuals and prostate cancer cells from N = 10 individuals were harvested from prostate tissue by laser capture microdissection, and gene expression determined. Data are the mean ± SEM of expression for each gene evaluated on the array, normalized as described in Methods. Lines denote 1.8 fold differences from the line of unity. Outlier genes are denoted, were determined as described in Methods, and associated Student's 2-sided P-values for differences between cancer and normal are depicted.
