# Supplementary material for: Genistein treatment duration effects biomarkers of cell motility in human prostate
Source: PLoS One. 2019 Mar 27;14(3):e0214078. doi: 10.1371/journal.pone.0214078 (PMC6436751; doi:10.1371/journal.pone.0214078)
Supplement: S2 Fig — (PDF) [file pone.0214078.s002.pdf]

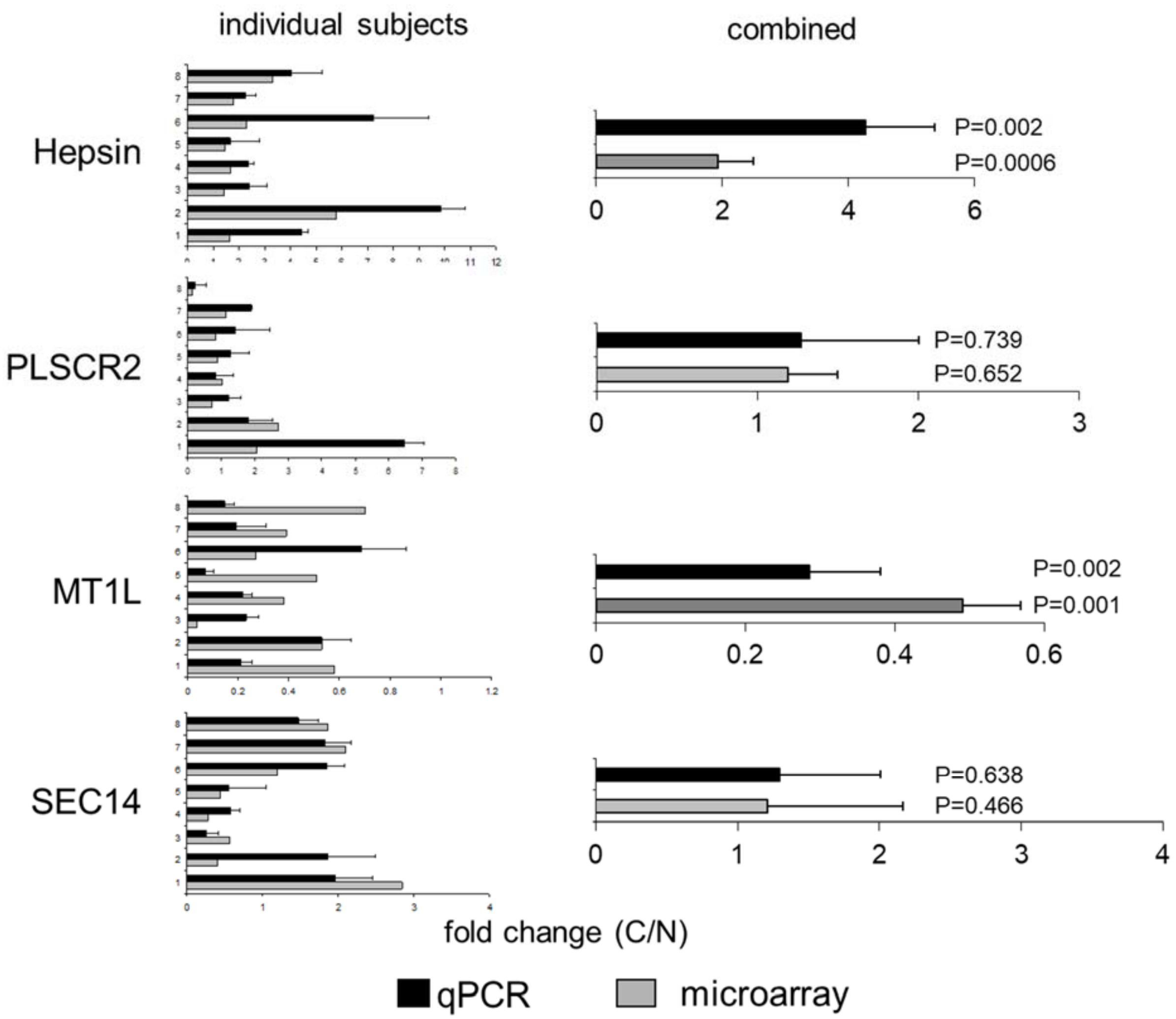

S2 Fig. Evaluation of gene expression by qRT/PCR. For each of four genes denoted, the left charts depict mean cancer/normal (C/N) ratio of gene expression for individual patients; the right charts are combined data. Gene expression for qRT/PCR was normalized to that of GAPDH, while that for microarray was Z normalized, as described in Methods. For individual subjects qRT/PCR was performed in replicates of N = 3, data are the mean ± SEM, microarray was performed once, N = 1, the resultant data depicted, and combined values are from N = 8 subjects,

data are the mean ± SEM. Student's 2-sided P-values for differences between cancer and normal are depicted.
