# Supplementary material for: Genistein treatment duration effects biomarkers of cell motility in human prostate
Source: PLoS One. 2019 Mar 27;14(3):e0214078. doi: 10.1371/journal.pone.0214078 (PMC6436751; doi:10.1371/journal.pone.0214078)
Supplement: S3 Fig — (PDF) [file pone.0214078.s003.pdf]

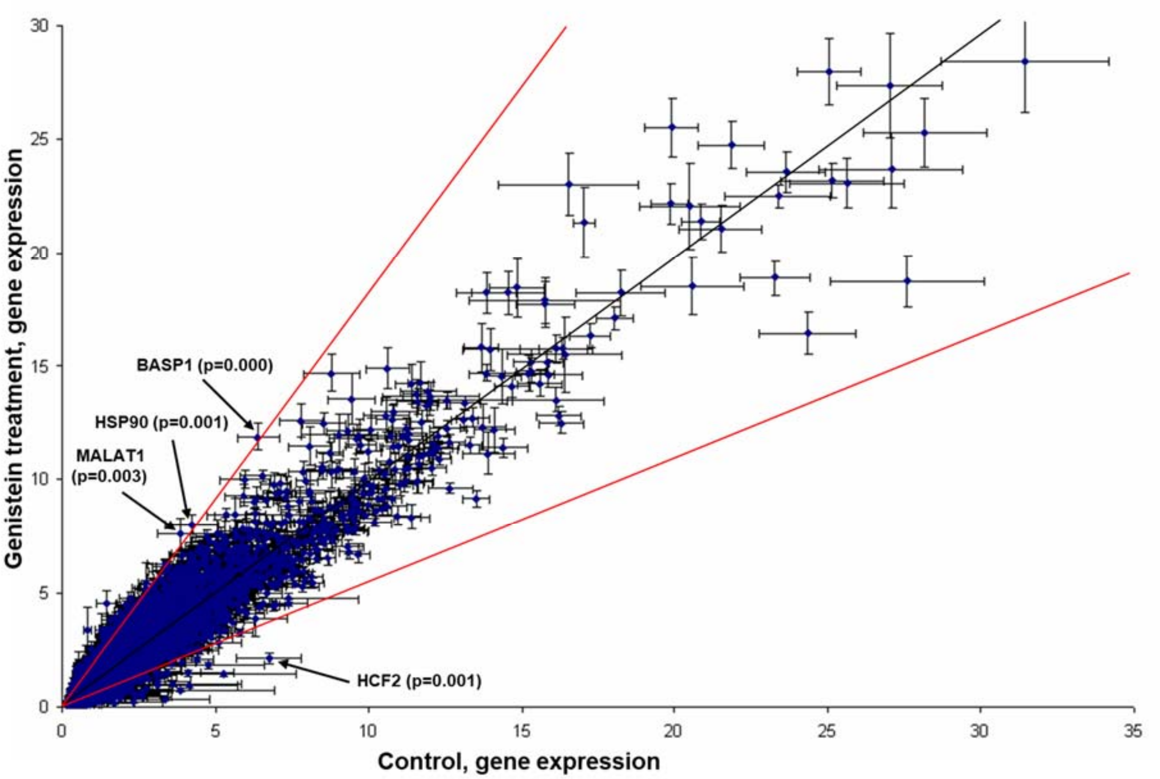

S3 Fig. Differences in gene expression in normal epithelial cells in human prostate tissue between genistein treated and control subjects. Gene expression was measured in normal prostate epithelial cells from N = 14 genistein treated and N = 14 control subjects, as in S1 Fig. Data are the mean ± SEM of expression for each gene measured on the array. Lines denote 1.8 fold differences from the line of unity. Outlier genes, as defined in Methods, are denoted.
