# Supplementary material for: Genistein treatment duration effects biomarkers of cell motility in human prostate
Source: PLoS One. 2019 Mar 27;14(3):e0214078. doi: 10.1371/journal.pone.0214078 (PMC6436751; doi:10.1371/journal.pone.0214078)
Supplement: S4 Fig — (PDF) [file pone.0214078.s004.pdf]

Table. List of all genes with P value </= 0.003

Gene symbol	GenBank accession	P-Value	mean Z score control	mean Z score genistein	Z score of highest group*	Ratio (largest/smallest)
BASP1	NM_006317	0.000008	6.40	11.89	11.89	1.86
HSP90	D87666	0.001259	4.25	8.02	8.02	1.89
MALAT1	AL050210	0.002622	3.88	7.59	7.59	1.96
HCF2	NM_000185	0.000754	6.74	2.11	6.74	3.19
NDUFAF3	AL049955	0.000182	3.34	6.26	6.26	1.87
SHC2	AB001451	0.000029	3.39	6.15	6.15	1.82
SRRM2	NM_016333	0.000335	2.72	5.22	5.22	1.92
RPL3	NM_000967	0.001032	2.73	5.14	5.14	1.88
SFPQ	NM_005066	0.001569	2.64	4.82	4.82	1.82
CEBPD	NM_005195	0.000250	2.25	4.77	4.77	2.11
MARCKS	AB020968	0.000449	2.60	4.69	4.69	1.80
ARL6IP5	NM_006407	0.000035	2.48	4.57	4.57	1.84
ZC3H11A	NM_014827	0.000014	2.08	4.50	4.50	2.16
ARID2	AB046777	0.000011	2.16	4.40	4.40	2.03
IL10RB	NM_000628	0.000632	2.32	4.40	4.40	1.90
MIEF1	NM_013298	0.001055	4.40	2.07	4.40	2.12
PHIP	NM_017934	0.000001	2.14	4.33	4.33	2.02
ARPC2	AF116702	0.000140	2.27	4.31	4.31	1.90
PDE8B	AF079529	0.000048	2.37	4.31	4.31	1.82
ABHD11	AF217971	0.000034	2.36	4.28	4.28	1.82
PART1	NM_016590	0.000778	2.28	4.26	4.26	1.87
CYTH1	NM_004762	0.000539	2.20	4.19	4.19	1.90
MLLT1	AL365410	0.001434	2.15	4.19	4.19	1.95
PSMA2	NM_002787	0.001004	2.18	4.17	4.17	1.91
SNRNP25	AK026593	0.000363	2.24	4.14	4.14	1.85
ANKRD17	AK025859	0.000105	2.21	4.04	4.04	1.83
FSTL1	NM_007085	0.000163	2.21	4.04	4.04	1.82
SPATS2L	NM_015535	0.000681	2.15	4.03	4.03	1.87
ZNF697	AK027019	0.000175	2.14	3.97	3.97	1.85
SATB1	NM_002971	0.000141	2.18	3.96	3.96	1.82
TRIM13	NM_005798	0.000104	2.06	3.87	3.87	1.88
ZNF613	AK023652	0.000196	2.11	3.81	3.81	1.81

* top 3% is Z score >/= 6.72

S4 Fig. List of all genes with P </= 0.003 for the difference between genistein and control.

The top four genes, BASP1, HSP90, MALAT1 and HCF2, meet the additional criteria to be identified as outlier genes.
