# Supplementary material for: Genistein treatment duration effects biomarkers of cell motility in human prostate
Source: PLoS One. 2019 Mar 27;14(3):e0214078. doi: 10.1371/journal.pone.0214078 (PMC6436751; doi:10.1371/journal.pone.0214078)
Supplement: S5 Fig — (PDF) [file pone.0214078.s005.pdf]

A

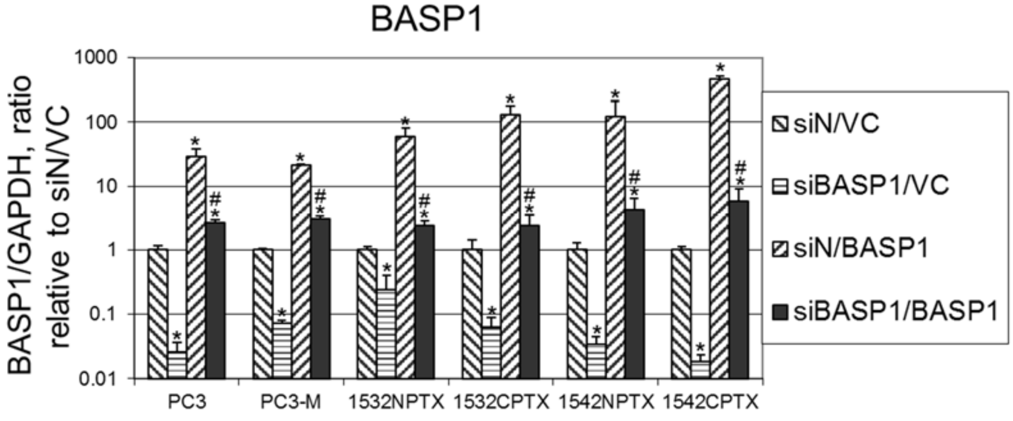
BASP1
BASP1/GAPDH, ratio relative to siN/VC
1000
100
10
1
0.1
0.01
PC3
PC3-M
1532NPTX
1532CPTX
1542NPTX
1542CPTX
siN/VC
siBASP1/VC
siN/BASP1
siBASP1/BASP1

B

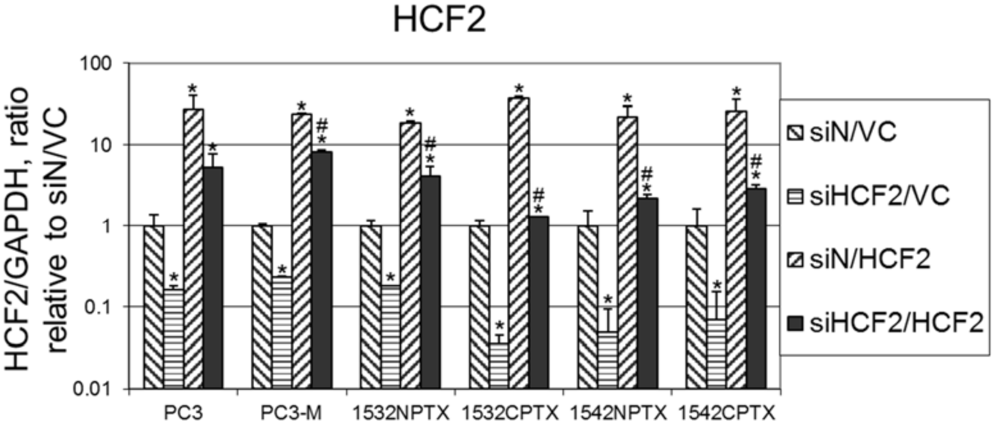
HCF2
HCF2/GAPDH, ratio relative to siN/VC
100
10
1
0.1
0.01
PC3
PC3-M
1532NPTX
1532CPTX
1542NPTX
1542CPTX
siN/VC
siHCF2/VC
siN/HCF2
siHCF2/HCF2

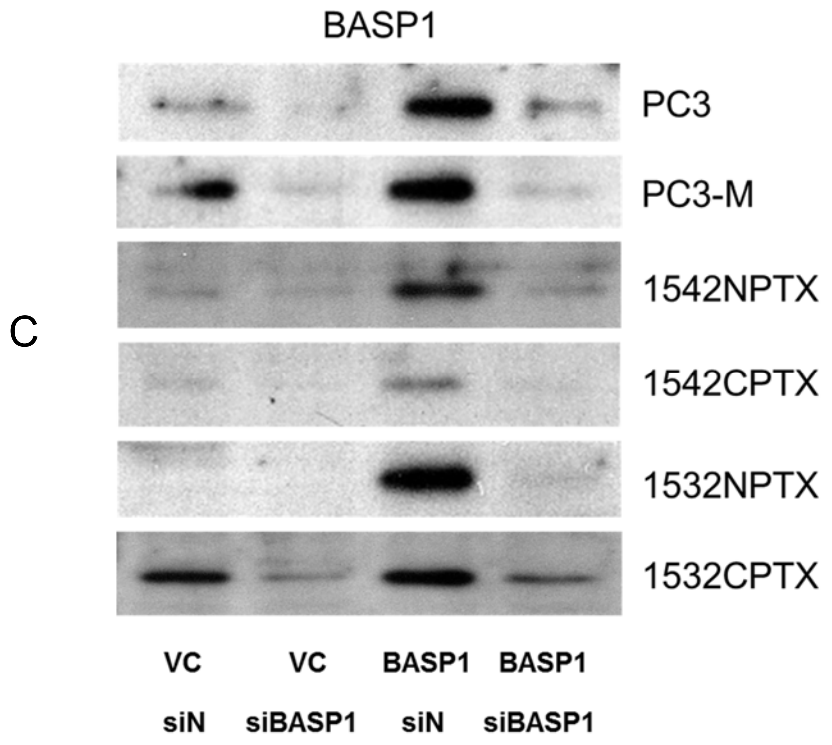
BASP1
C
PC3
PC3-M
1542NPTX
1542CPTX
1532NPTX
1532CPTX
VC
siN
VC
siBASP1
BASP1
siN
BASP1
siBASP1

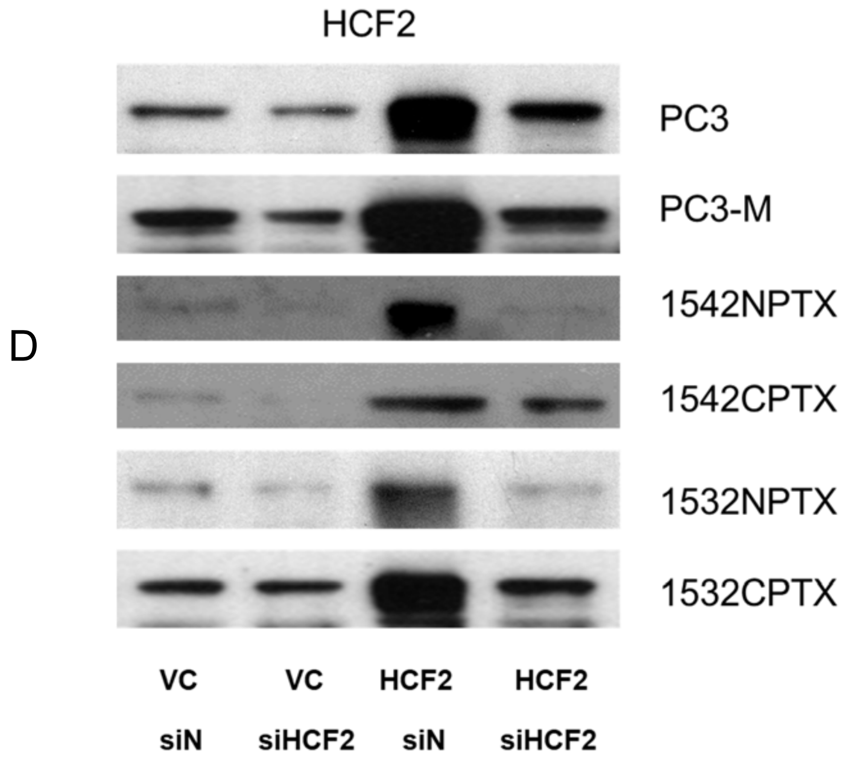
HCF2
D
PC3
PC3-M
1542NPTX
1542CPTX
1532NPTX
1532CPTX
VC
siN
VC
siHCF2
HCF2
siN
HCF2
siHCF2

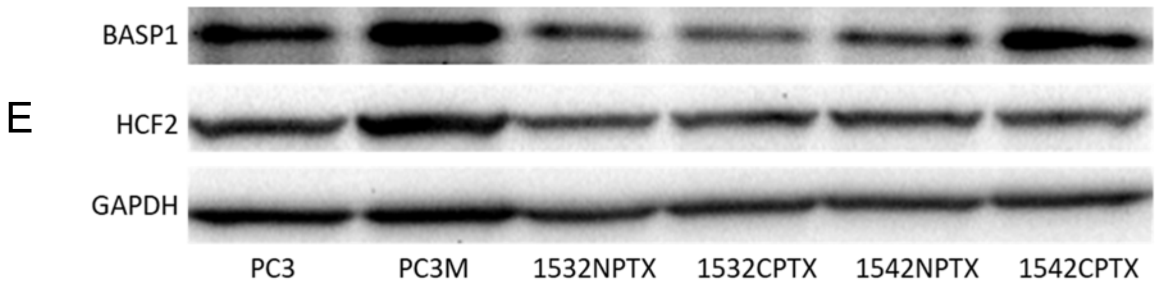
E
BASP1
HCF2
GAPDH
PC3
PC3M
1532NPTX
1532CPTX
1542NPTX
1542CPTX

S5 Fig. Effect of BASP1 and HCF2 knockdown and overexpression on respective transcript and protein levels. The denoted human prostate cell lines were transfected with gene-specific siRNA, non-targeting siRNA (siN), with gene-specific vector and/or with empty vector control (VC), as indicated. **A and B**) Expression of transcripts was measured by qRT/PCR. Data are the mean ± SD (replicates of N =2) level of expression, normalized to GAPDH, and expressed relative to that of control cells (i.e., siN/VC), which were set to 1.0; * $P < 0.05$ compared to siN/VC, # $P < 0.05$ for comparison between siN/gene-specific vector and gene-specific siRNA/gene-specific vector. (Note, the comparison between siN/HCF and siHCF/HCF for PC3 cells was a trend, with two sided $P = 0.07$; one sided t-text P value 0.04) **C and D**) Protein expression was measured by Western blot. Representative blots from equally loaded lanes are depicted. **E.** comparison of BASP1 and HCF2 protein expression in native cell lines measured by Western blot.
