# Supplementary material for: Genistein treatment duration effects biomarkers of cell motility in human prostate
Source: PLoS One. 2019 Mar 27;14(3):e0214078. doi: 10.1371/journal.pone.0214078 (PMC6436751; doi:10.1371/journal.pone.0214078)
Supplement: S6 Fig — (PDF) [file pone.0214078.s006.pdf]

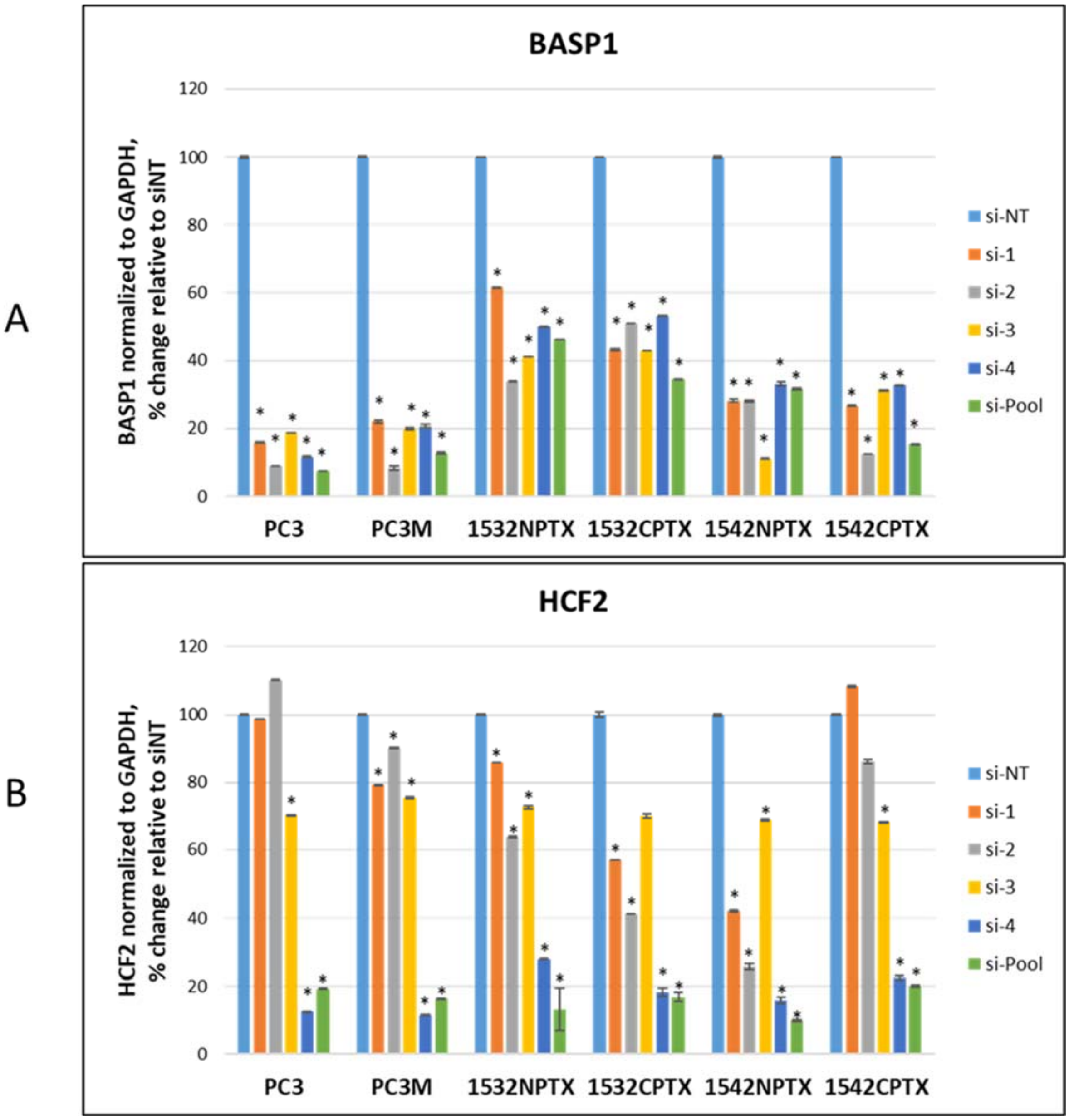

S6 Fig. Effect of individual siRNA species on target gene transcript levels. The denoted human prostate cell lines were transfected with gene-specific siRNA Pools, the de-convoluted individual gene-specific siRNA from the pool or non-targeting siRNA (siNT), as indicated. Gene expression for qRT/PCR was normalized to that of GAPDH, as described in Methods. Data are the mean ± SEM (replicates of N =3) expressed relative to that of control (siNT), which were set

to 100; * $P < 0.05$ for 2-sided Student's t-test compared to control. **A)** Efficacy of BASP1 siRNA (Dharmacon: si-1: J-019008-06, si-2: J-019008-07, si-3: J-019008-08, si-4: J-019008-09, si-Pool: J-019008-00 and si-NT: D-001810-10). **B)** Efficacy of HCF2 siRNA (Dharmacon: si-1: J-009540-05, si-2: J-009540-06, si-3: J-009540-07, si-4: J-009540-08, si-Pool: J-009540-00 and si-NT: D-001810-10)
