# Supplementary material for: Genistein treatment duration effects biomarkers of cell motility in human prostate
Source: PLoS One. 2019 Mar 27;14(3):e0214078. doi: 10.1371/journal.pone.0214078 (PMC6436751; doi:10.1371/journal.pone.0214078)
Supplement: S7 Fig — (PDF) [file pone.0214078.s007.pdf]

A

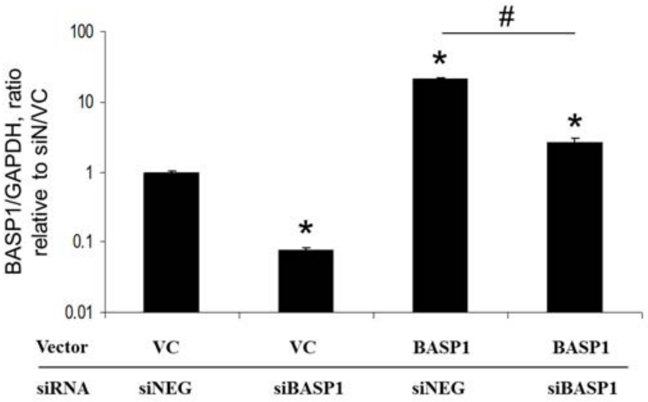
BASP1/GAPDH, ratio relative to siN/VC
100
10
1
0.1
0.01
#
*
Vector VC VC BASP1 BASP1
siRNA siNEG siBASP1 siNEG siBASP1

B

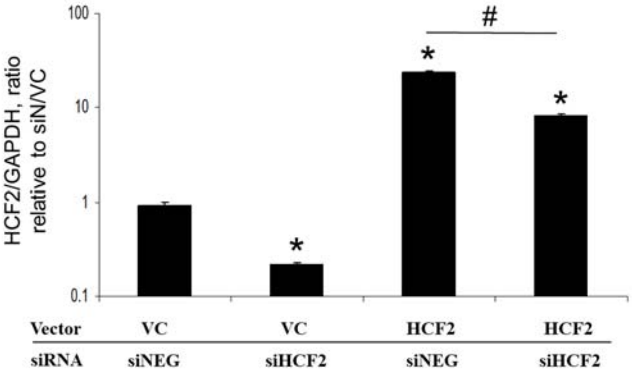
HCF2/GAPDH, ratio relative to siN/VC
100
10
1
0.1
#
*
Vector VC VC HCF2 HCF2
siRNA siNEG siHCF2 siNEG siHCF2

C

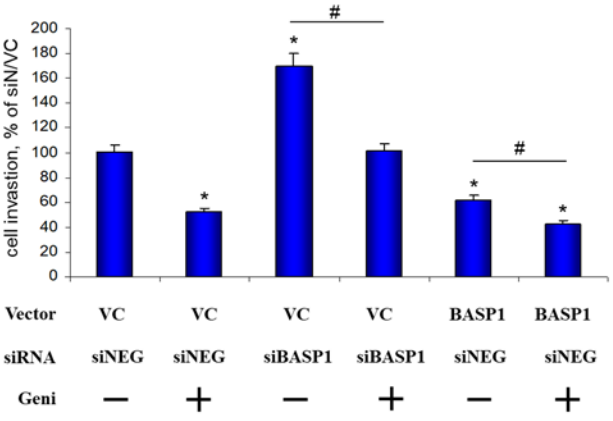
cell invastion, % of siN/VC
200
180
160
140
120
100
80
60
40
20
0
#
*
Vector VC VC VC VC BASP1 BASP1
siRNA siNEG siNEG siBASP1 siBASP1 siNEG siNEG
Geni − + − + − +

D

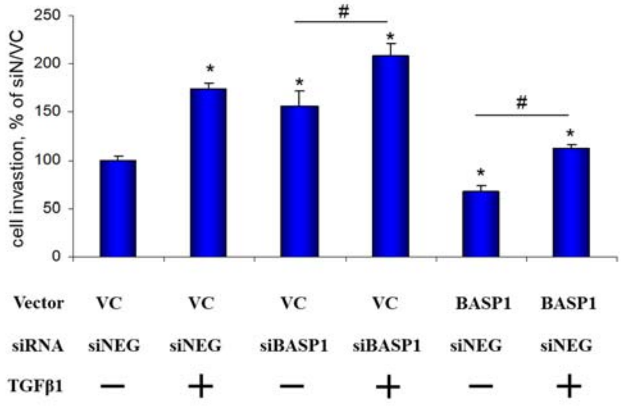

cell invasion, % of siN/VC
250
200
150
100
50
0
#
#
Vector VC VC VC VC BASP1 BASP1
siRNA siNEG siNEG siBASP1 siBASP1 siNEG siNEG
TGFβ1 − + − + − +

E

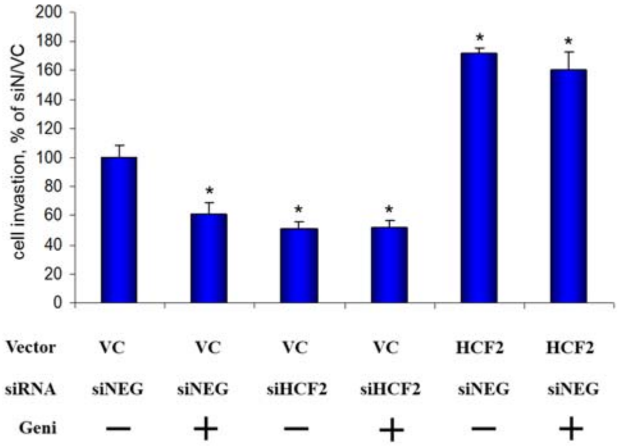

cell invasion, % of siN/VC
200
180
160
140
120
100
80
60
40
20
0
Vector VC VC VC VC HCF2 HCF2
siRNA siNEG siNEG siHCF2 siHCF2 siNEG siNEG
Geni − + − + − +

F

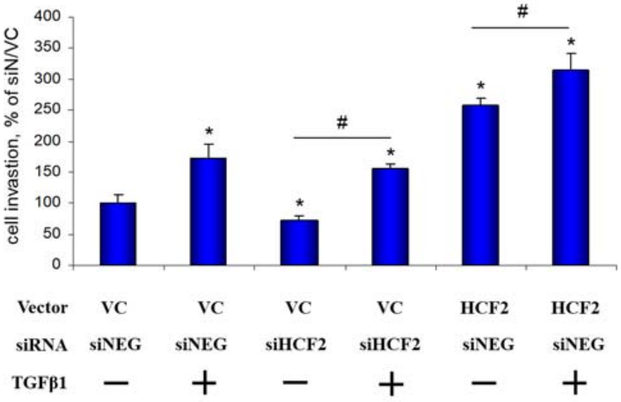

cell invasion, % of siN/VC
400
350
300
250
200
150
100
50
0
#
#
Vector VC VC VC VC HCF2 HCF2
siRNA siNEG siNEG siHCF2 siHCF2 siNEG siNEG
TGFβ1 − + − + − +

S7 Fig. The effect of BASP1 and HCF2 on human prostate cell invasion and genistein efficacy. PC3-M cells were transfected as in S3 Fig, treated with genistein or TGFβ as indicated. **A and B**) Expression of transcripts was measured by qRT/PCR, and data expressed as in S3 Fig. **C-F**) Cell invasion. Data from Boyden chamber cell invasion assays are the mean ± SEM (replicates of N =4) cell invasion, expressed as the percent of control cells (i.e., siN/VC). *denotes P </= 0.05 for 2-sided Student's t-test compared to control; # denotes P </= 0.05 for conditions denoted by line.
